# A survey on the awareness of a sleep-medication formulary in community health care and its influence on prescribing behavior

**DOI:** 10.1186/s40780-025-00521-7

**Published:** 2025-12-08

**Authors:** Akira Ishikawa, Ryosuke Mizumura, Satoru Utsunomiya, Moriyuki Ito, Yohei Kawasaki, Yuki Shiko, Maiko Osawa, Hideki Makabe, Koji Matsuo

**Affiliations:** 1 Department of Pharmacy, Saitama Medical University Hospital, 38 Morohongo, Moroyama, Iruma, Saitama, 350-0495 Japan; 2 Department of Psychiatry, Saitama Medical University Hospital, Moroyama, Saitama, Japan; 3 Department of Biostatistics, Graduate School of Saitama Medical University, Moroyama, Saitama, Japan; 4 Saitama Medical University International Medical Center, Hidaka, Saitama, Japan

**Keywords:** Formulary, Sleep medication, Prescribing behavior, Community healthcare, Physician awareness.

## Abstract

**Background:**

Despite the increasing emphasis on safe prescribing, the impact of local formularies on physician behavior remains underexplored in community healthcare settings. To promote safer pharmacological practices, our institution implemented a hospital-originated sleep-medication formulary in January 2022, which was subsequently disseminated to other local medical institutions in early 2023. This study assessed physicians’ awareness of this sleep-medication formulary, and its influence on prescribing behavior in local community healthcare settings.

**Methods:**

A questionnaire survey was distributed via a regional medical-cooperation newsletter and responses were received from 84 physicians.

**Results:**

Of the 84 physicians, 18% were aware of the formulary. Physicians in the awareness group were more likely to prescribe the recommended medications, particularly orexin receptor antagonists, and reported significantly less difficulty in initiating hypnotic therapy. Logistic regression analysis revealed a significant association between formulary awareness and reduced difficulty in prescribing a suitable hypnotic (odds ratio: 0.24; 95% CI: 0.073–0.78).

**Conclusions:**

Local formularies may serve as valuable tools in clinical decision-making and promote safer and more consistent prescribing practices. Strategies such as physician education, concise formulary reference sheets or physician-oriented pocket references, and the development of patient-friendly information materials are warranted to support broader implementation.

**Trial registration:**

Not applicable.

## Background

Insomnia is highly prevalent among older adults, and the use of sleep medications continues to increase [1]. However, conventional hypnotics such as benzodiazepines are associated with adverse events, including falls, delirium, and cognitive impairment. The 2023 American Geriatrics Society Beers Criteria^®^ recommends that these medications generally be avoided in older adults [2].

In response to these safety concerns, many institutions have developed formularies to guide safer pharmacological practices by clearly designating recommended medications. A formulary is defined as a structured list or set of guidelines for medication selection and use, based on comprehensive evaluations of efficacy, safety, and cost-effectiveness. Formularies were initially implemented to standardize and optimize drug therapy in hospital settings. More recently, attention has turned to their application in broader community settings—including outpatient clinics and home healthcare. These are referred to as “regional formularies” [3].

Given the aging population and expansion of community-based integrated care systems, consistent prescribing practices and effective information-sharing among healthcare providers have become increasingly important. In January 2022, our institution introduced a sleep-medication formulary primarily for in-hospital use. To promote collaboration beyond the hospital setting, we subsequently disseminated the formulary to local medical institutions through publications such as the *Regional Medical Cooperation News*, lectures, and conference presentations.

Although regional formularies are gradually being developed, their broader adoption in Japan requires consensus-building among various stakeholders, including medical, dental, and pharmaceutical associations, as well as administrative bodies. In addition, operational flexibility is required to adapt to local healthcare contexts. The Ministry of Health, Labour and Welfare (MHLW) has emphasized the importance of regional formularies, advocating for community-wide sharing of standardized treatment protocols and encouraging the expansion of institutional efforts to regional levels [4].

While the use of formularies in hospital settings is well-established, their role in community healthcare remains underexplored [4, 5]. In particular, empirical evidence is lacking on physicians’ awareness of regional formularies and how such awareness may influence prescribing behavior in real-world settings. To address this gap, we conducted a questionnaire-based survey of physicians at local medical institutions, focusing on awareness of a hospital-originated formulary disseminated to the community. Approximately 18 months after the initial launch and dissemination of our sleep-medication formulary, the present study assessed physicians’ awareness and examined its influence on prescribing behavior. Furthermore, the perceived practicality of the formulary and barriers to broader adoption were explored.

## Methods

### Study design and respondents

This cross-sectional observational study aimed to evaluate the level of awareness of a hospital-originated sleep-medication formulary, created at Saitama Medical University Hospital and subsequently disseminated to local medical institutions, and its influence on prescribing practices in the context of community healthcare. The target population comprised physicians working at medical institutions, who subscribed to the *Community Medical Cooperation News* issued by Saitama Medical University Hospital. Responses were provided by individual physicians on a voluntary basis, not necessarily by institutional representatives. As respondents were drawn only from a list of newsletter subscribers, they may not be representative of all community physicians. The choice to respond to the survey was voluntary, and informed consent was obtained following a review of the study’s explanation document. The survey was conducted between July 26 and August 26, 2024.

### Dissemination of the formulary

Activities to raise awareness of the formulary were conducted during the 18 months following the formulary’s launch. Dissemination of the formulary was promoted through multiple initiatives, beginning with the publication of the methodology for formulary development [6]. Additional strategies included publication in the *Community Medical Cooperation News*, presentations at academic conferences, lectures for physicians, and, within the hospital, e-learning programs designed for physicians and multidisciplinary staff.

### Questionnaire content

The questionnaire was used to collect demographic information such as years of clinical experience, type of medical institution, and geographic area. Additional items assessed awareness of the formulary, prescribing behavior, and suggestions for ways to improve the formulary and its use. The questionnaire items are summarized in Table 1.

**Table 1:** Questionnaire items used in the survey

No.	Item	Response options
1	Participation in this survey	Agree / Disagree
2	Years of clinical experience	< 5 years; 5–10 years; 10–15 years; 15–20 years; >20 years
3	Type of medical institution	Clinic; Hospital (< 200 beds; 200–399 beds; ≥400 beds)
4	Region of medical institution	Hanno–Hidaka; Iruma; Sayama; Chichibu; Hiki; Sakado–Tsurugashima; Other
5	Main department of practice	Free entry
6	Have you ever experienced difficulty in selecting an initial hypnotic?	Yes / No
7	Sleep medications you most frequently prescribe (choose one)	Benzodiazepines (e.g., flunitrazepam, triazolam, brotizolam); Non-benzodiazepines (e.g., zopiclone, zolpidem, eszopiclone); Orexin receptor antagonists (e.g., lemborexant, suvorexant); Melatonin receptor agonists (e.g., melatonin, ramelteon); Other (free entry)
8	Are you aware of Saitama Medical University Hospital’s sleep-medication formulary?	Yes (go to Q9) / No (go to Q16)
9	How did you become aware of the formulary? (multiple answers allowed)	Community Medical Cooperation News; Colleagues; Other medical institutions (e.g., workplaces, physician acquaintances); Lectures/research papers; Other
10	After becoming aware of the formulary, did you start prescribing the recommended drugs?	Yes / No (skip to Q14)
11	If “Yes” to Q10: In approximately what percentage of your patients do you prescribe the recommended drugs?	1–25%; 26–50%; 51–75%; 76–100%
12	Among the recommended drugs, which do you prescribe most frequently?	Lemborexant; Eszopiclone; Ramelteon; Zolpidem
13	Among the recommended drugs, which do you prescribe second most frequently?	Lemborexant; Eszopiclone; Ramelteon; Zolpidem
14	Would you recommend the formulary to others?	Yes / No / Unsure
15	If you have any suggestions or concerns about the formulary, please describe.	Free text
16	(If answered “No” to Q8) Would you like to use the formulary in the future?	Yes / No / Unsure
17	If you have any suggestions or concerns about the formulary, please describe.	Free text

## Data-collection procedures

The questionnaire was distributed as an attachment to the *Community Medical Cooperation News*. The responses were collected using the following two methods:

1. Online submission using Google Forms accessed via a QR code printed in the newsletter.
2. Fax submission of a completed paper-based form to the Department of Pharmacy, Saitama Medical University Hospital.

### Statistical analysis

Incomplete or unclear responses were excluded from the analysis of relevant items. All statistical analyses were performed using JMP software version 17 (SAS Institute Inc., Cary, NC, USA). Continuous variables are presented as the mean ± standard deviation (SD) and categorical variables as counts and percentages (%).

The differences between the two groups were compared using t-tests for continuous variables and chi-square tests for categorical variables, with Fisher’s exact test applied when the expected cell counts were less than five. Awareness of the formulary was categorized into two groups:

- Aware group: physicians who reported being aware of the formulary
- Non-aware group: physicians who were unaware of the formulary

To identify independent predictors of reduced difficulty in initial hypnotic selection for prescriptions, logistic regression analysis was performed using awareness of the formulary, years of clinical experience, and institution type as explanatory variables.

Open-ended responses were subjected to content analysis. Common themes were extracted and categorized to identify frequently mentioned issues and areas for improvement. A p-value < 0.05 was considered statistically significant. Open-ended responses were reviewed and categorized into six thematic groups. Then, the number of responses in each category was counted and summarized in a table.

### Ethical considerations

This study was approved by the Ethics Review Committee of Saitama Medical University (Approval No.: 2023-027). All respondents received detailed information about the study’s purpose, procedures, and confidentiality measures. Informed consent was obtained through written agreement or electronic confirmation via an online form in accordance with the ethical research guidelines.

## Results

### Response rate

We distributed the newsletter containing the questionnaire, one copy per institution, to approximately 1,500 medical institutions, including both hospitals and clinics, and obtained responses from 84 physicians. As each institution received only one copy, the actual number of physicians who viewed the questionnaire was unknown. Moreover, multiple physicians within a facility could respond; therefore, the unit of analysis was the individual respondent.

### Respondent characteristics

Table 2 shows the distribution of the respondents’ basic characteristics, including the breakdown by type of medical institution. Most had 20 or more years of clinical experience (79%), while only 1% had fewer than 5 years. Most of the respondents worked in clinics (74%), followed by those in hospitals with fewer than 200 beds (21%). The most common medical specialty was internal medicine, accounting for 71% of the responses.

**Table 2:** Basic characteristics of responding physicians

Attribute	Category	*n* (%)
Years of clinical experience	< 5 years	1 (1.2)
	5 –< 10 years	4 (4.8)
	10 –< 15 years	6 (7.1)
	15 –< 20 years	7 (8.3)
	≥ 20 years	66 (78.6)
Type of medical institution	Clinic	62 (73.8)
	Hospital (< 200 beds)	18 (21.4)
	Hospital (200 –< 400 beds)	3 (3.6)
	Hospital (≥ 400 beds)	1 (1.2)
Medical specialty (multiple responses)	Internal medicine	61
	Surgery	10
	Psychiatry	7
	Obstetrics & gynecology	4
	Pediatrics	2
	Other	2

**Note.** Percentages are calculated based on the total number of respondents (*N* = 84). Respondents could select only one institution type but were allowed to select multiple specialties. “Clinic” refers to outpatient-only facilities, while “Hospital” includes all institutions providing inpatient care

### Trends in sleep-medication prescribing

The sleep-medication formulary used at our facility, from which respondents selected recommended hypnotics, is summarized in Table 3.

**Table 3:** Sleep-medication formulary at Saitama Medical University Hospital

For adults		
Recommendation	Drug name	Recommended dose
1st-line	Lemborexant (Dayvigo^®^) 5 mg	5 mg (may adjust); do not exceed 10 mg
2nd-line	Eszopiclone 1 mg, 2 mg	2 mg (may adjust); do not exceed 3 mg
3rd-line	Ramelteon (Rozerem^®^) 8 mg	8 mg
	Zolpidem tartrate 5 mg, 10 mg	5–10 mg; do not exceed 10 mg
For elderly (≥ 65 years) or patients at risk of delirium		
1st-line	Lemborexant (Dayvigo^®^) 2.5 mg, 5 mg	5 mg (may adjust); do not exceed 10 mg
2nd-line	Ramelteon (Rozerem^®^) 8 mg	8 mg
3rd-line	Eszopiclone 1 mg, 2 mg	1 mg (may adjust); do not exceed 2 mg

Dosages are expressed as recommended daily doses. “1st-line,” “2nd-line,” and “3rd-line” indicate the prioritization of hypnotics within the formulary. Recommended doses for elderly patients (≥ 65 years) or those at risk of delirium are shown separately. Brand names are indicated for drugs prescribed in branded forms at our hospital (Dayvigo^®^, Rozerem^®^); other drugs were generally prescribed as generics.

**Fig. 1:**
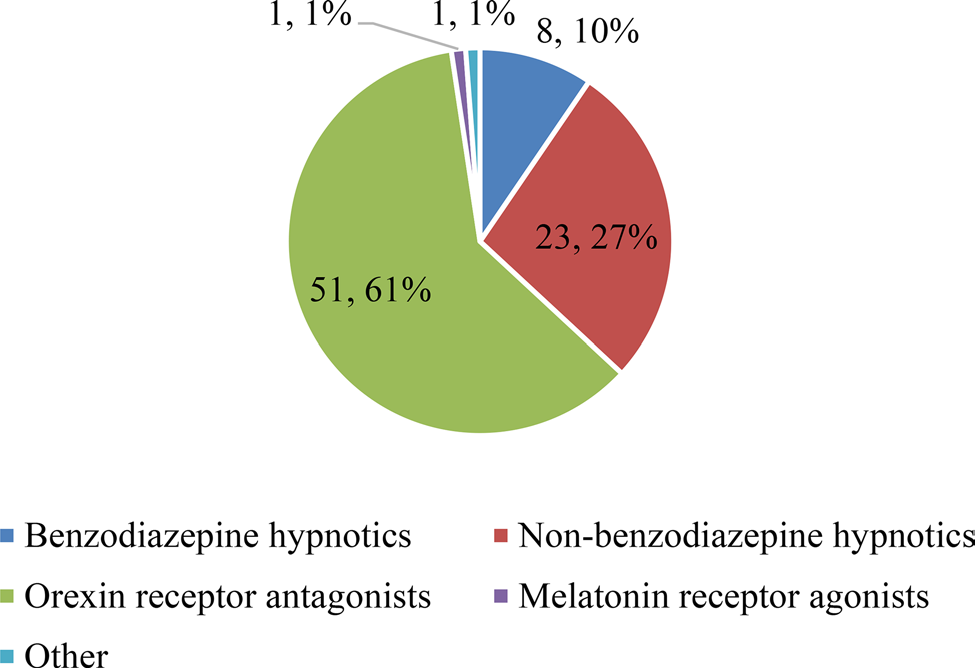
Distribution of frequently prescribed sleep medications

The sleep medications most frequently prescribed by the respondents were orexin receptor antagonists (61%), followed by non-benzodiazepines (27%) and benzodiazepines (10%). Melatonin receptor agonists and other agents accounted for 1% of the prescriptions (Fig. 1).

The most frequently prescribed medications were orexin receptor antagonists (61%), followed by non-benzodiazepines (27%) and benzodiazepines (10%). Melatonin receptor agonists and other agents accounted for 1% of prescriptions. Percentages indicate the proportion of respondents who reported prescribing each category.

### Awareness of the formulary

Of the 84 respondents, 15 (18%) were aware of the formulary (aware group), whereas 69 (82%) were not (non-aware group).

### Use of recommended medications among the awareness group

In the aware group, 87% reported that they had begun prescribing the recommended medications. The frequency of use was as follows:

- 1–25 % of patients: 8 %
-  26–50 %: 0 %
- 51–75 %: 54 %
- 76–100%: 38%

The most frequently prescribed recommended drug was lemborexant (80%), followed by zolpidem (27%), ramelteon (27%), and eszopiclone (13%).

### Association between formulary awareness and physician attributes

Table 4 summarizes the association between formulary awareness and physician attributes, including years of clinical experience and type of medical institution.

**Table 4:** Association between physician attributes and formulary awareness (*n* = 84)

Attribute	Category	Aware (*n* = 15)	Non-aware (*n* = 69)
Years of clinical experience	< 5 years	0 (0.0%)	1 (1.4%)
	5 –< 10 years	1 (6.7%)	3 (4.3%)
	10 –< 15 years	0 (0.0%)	6 (8.7%)
	15 – < 20 years	2 (13.3%)	5 (7.2%)
	≥ 20 years	12 (80.0%)	54 (78.3%)
Type of medical institution	Clinic	10 (66.7%)	52 (75.4%)
	Hospital (< 200 beds)	4 (26.7%)	14 (20.3%)
	Hospital (200 – <400 beds)	1 (6.7%)	2 (2.9%)
	Hospital (≥ 400 beds)	0 (0.0%)	1 (1.4%)

Values are expressed as number (%). No statistically significant differences in formulary awareness were observed according to clinical experience or type of medical institution (*p* > 0.05)

There were no statistically significant differences in formulary awareness according to years of experience or type of medical institution (hospital vs. clinic) (*p* > 0.05), suggesting that awareness was not strongly associated with these characteristics.

### Awareness of the formulary and difficulty in selecting an initial hypnotic

**Fig. 2:**
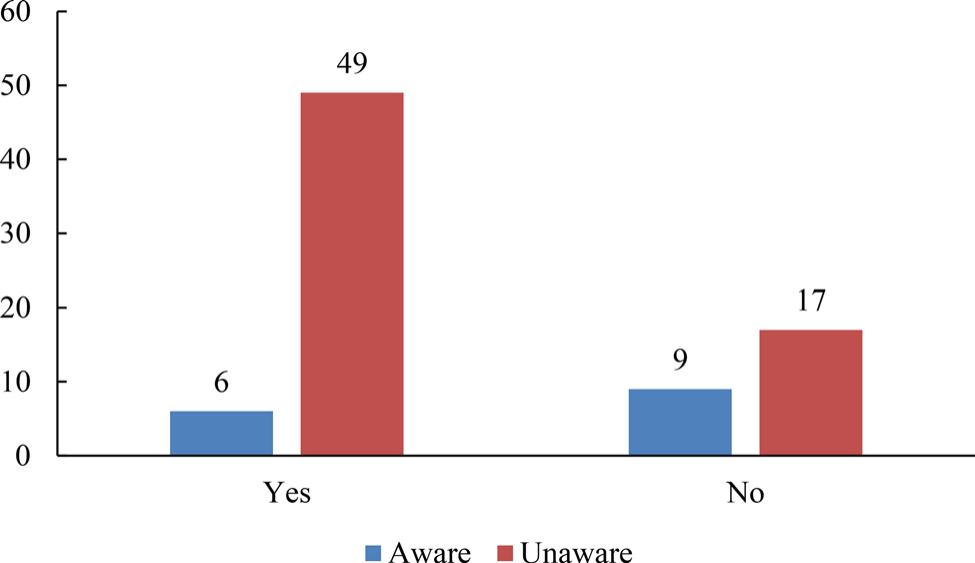
Association between formulary awareness and difficulty in selecting an initial hypnotic

Among physicians in the aware group, 40% reported difficulty in selecting an initial hypnotic, compared with 74% in the non-aware group. This difference was statistically significant according to Fisher’s exact test (*p* = 0.0152) (Fig. 2).

The y-axis indicates the percentage of respondents within each group. The aware group included physicians aware of the formulary (*n* = 15), while the non-aware group included those unaware of the formulary (*n* = 66), with three physicians not responding to this question. A significantly higher proportion of physicians in the non-aware group reported difficulty in selecting an initial hypnotic compared with the aware group (*χ²* test, *p* < 0.05). Fisher’s exact test produced similar results.

Logistic regression analysis was conducted using formulary awareness, years of clinical experience, and institution type as independent variables. Awareness of the formulary was significantly associated with reduced difficulty in selecting an initial hypnotic to prescribe (odds ratio, 0.24; 95% CI, 0.073–0.78; *P* = 0.0178). Neither years of experience nor institution type showed a significant association (Table 5).

**Table 5:** Multivariate logistic regression results

Variable	Odds Ratio	95% CI (Lower)	95% CI (Upper)	*p*-value
Formulary awareness (aware vs. unaware)	0.24	0.073	0.78	0.0178
Years of clinical experience (≥ 15 years vs. < 15 years)	0.85	0.193	3.73	0.8278
Type of medical institution (clinic vs. hospital)	1.35	0.445	4.08	0.5979

CI: confidence interval; OR: odds ratio. Multivariate logistic regression was conducted to examine the factors associated with perceived difficulty in selecting an initial hypnotic. “Aware” refers to physicians aware of the formulary; “Unaware” refers to those unaware.Statistical significance was defined as p < 0.05.Statistical significance was defined as *p* < 0.05

### Awareness and prescribing patterns

Table 6 summarizes the prescribing patterns of the aware and non-aware groups. Orexin receptor antagonists were the most frequently prescribed in both groups, but the proportion was higher in the aware group. The chi-squared test showed no statistically significant difference between the two groups (*p* = 0.242)

**Table 6:** Prescribing patterns of sleep medications by formulary awareness

Drug category	Aware group (*n* = 15)	Non-aware group (*n* = 69)
Orexin receptor antagonists	86.7%	55.1%
Non-benzodiazepines	6.7%	31.9%
Benzodiazepines	6.7%	10.1%
Melatonin receptor agonists	0.0%	1.4%
Other	0.0%	1.4%

Values are expressed as percentages of respondents in each group. “Aware group” refers to physicians aware of the formulary; “Non-aware group” refers to those unaware

### Sources of formulary awareness

**Fig. 3:**
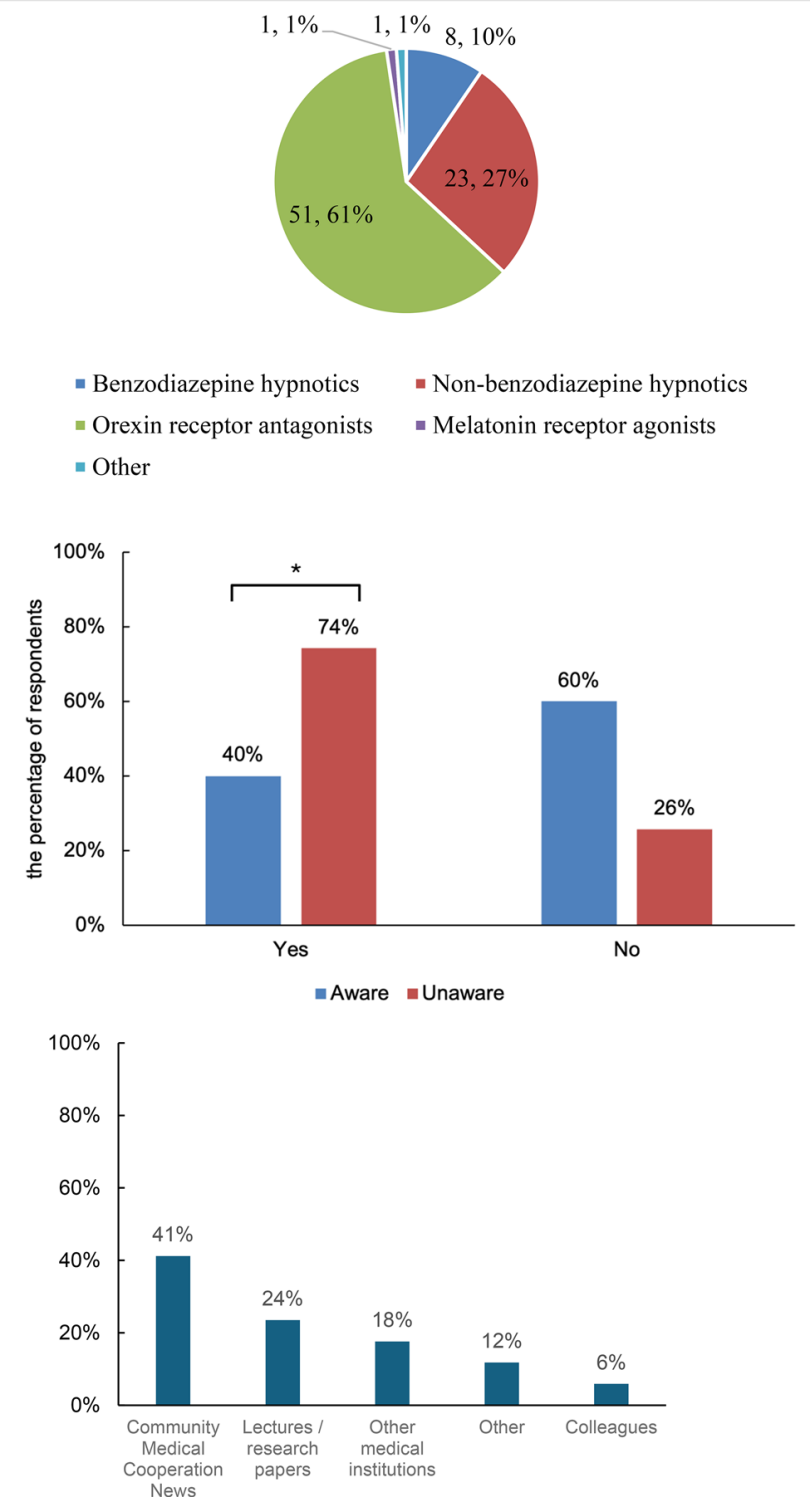
Sources of formulary awareness. The distribution of information sources reported by physicians aware of the formulary

Among those who were aware of the formulary, the Community Medical Cooperation News was the most frequently cited source (41%, *n* = 7), followed by lectures and research papers (23%, *n* = 4), other medical institutions (18%, *n* = 3), other sources (12%, *n* = 2), and colleagues (6%, *n* = 1). These results are shown in Fig. 3.

### Content analysis of open-ended responses

Content analysis of the 22 open-ended responses identified six thematic categories (Table 7).

**Table 7:** Categorized themes from open-ended responses (*n* = 22)

Category	Example comments	*n*
Fairness of description	“Use group names instead of brand names.”	2
Formulary description fairness	“The formulary considers drug cost and fall/delirium risk.”	3
Drug-specific efficacy and side effects	“Lemborexant sometimes less effective in BZD users”; “Zolpidem should not be recommended.”	5
Education needs	“Pamphlets for patient education are needed.”	3
Prescribing challenges	“Difficult to persuade long-term BZD users to switch.”	3
Miscellaneous/Other	“Include Kampo medicines as an option”; “No specific opinion.”	6

Free-text comments from respondents were grouped into thematic categories. Representative examples and the number of responses in each category are shown

The most frequent comments related to drug-specific efficacy and side effects (*n* = 5) and comments in the “Miscellaneous/other” category (*n* = 6), followed by issues such as education needs (*n* = 3) and prescribing challenges (*n* = 3). Representative comments included requests for more education of general practitioners, the suggestion that patient education materials such as pamphlets would be useful, and concerns that patients strongly resist switching from long-term benzodiazepines.

These themes highlight the potential barriers to broader adoption and suggest concrete areas for future improvement.

## Discussion

This study investigated awareness of a hospital-originated sleep-medication formulary, created at Saitama Medical University Hospital and disseminated to local institutions, and its impact on prescribing behavior among community physicians approximately 18 months after its implementation. Awareness was actively promoted during this period through various initiatives, as detailed in the Methods section. Despite a low overall awareness rate (18%), physicians who were aware of the formulary were significantly more likely to prescribe the recommended medications, particularly orexin receptor antagonists, and reported less difficulty in selecting an initial hypnotic. Logistic regression analysis confirmed that awareness of the formulary was significantly associated with reduced difficulty in selecting a medication to prescribe independent of clinical experience or institutional setting. These findings suggest that formularies may serve as valuable clinical decision-support tools, facilitating physicians’ prescribing process.

However, among respondents in the aware group, the most common prescribing frequency for the recommended drugs was 51–75% rather than the recommended 76–100%. This pattern may reflect real-world challenges, such as physicians’ reluctance to switch long-term benzodiazepine users. It may also indicate that, for some patients, alternative hypnotics were considered more appropriate. By contrast, non-benzodiazepines were more common in the non-aware group, likely reflecting conventional prescribing patterns. Benzodiazepines were prescribed at low rates overall but slightly more often in the non-aware group, indicating that formulary awareness may help promote safer prescribing trends.

Prescribing hypnotics often involves selecting from a wide variety of pharmacological classes while considering patient-specific factors, including comorbidities and concurrent medications. Previous research has indicated that non-psychiatrists tend to prioritize safety, often choosing melatonin receptor agonists despite their lower perceived efficacy [7]. This dilemma can lead to uncertainty in drug selection. Our findings are consistent with these previous surveys, including a national study by the MHLW that highlighted physicians’ uncertainty and heterogeneitywhen initially prescribing hypnotics [5]. Consistent with prior findings, the free-text responses in our study revealed concerns about side effects and risks, such as delirium and falls [5, 7]. A well-structured formulary that clearly specifies the recommended medications may help non-specialist physicians make informed, evidence-based prescribing decisions and alleviate clinical anxiety.

While several physicians expressed support for the efficacy and safety of the recommended medications, others raised practical challenges in implementing the formulary. Notably, 40% of physicians in the aware group still reported difficulty in selecting an initial hypnotic, indicating that awareness alone did not fully resolve prescribing challenges. For instance, physicians have reported that long-term benzodiazepine users are often resistant to switching medications, citing dissatisfaction or a fear of change. Comments such as “We try to prescribe appropriately, but patients often strongly resist” and “A patient-friendly pamphlet would help explain the change” highlight the need for patient education to facilitate appropriate use.

To address these challenges, the development and dissemination of patient-focused educational materials, such as informational pamphlets or medication-guidance sheets, may be effective. Some of the free-text responses in this study specifically suggested the need for such tools, noting that patients often resist switching from long-term benzodiazepines and that explanatory materials would facilitate acceptance. In addition, concise formulary reference sheets or physician-oriented pocket references could support general practitioners, who often have limited consultation time, by providing quick guidance on recommended options and their rationale. The importance of such educational and awareness-raising activities has also been highlighted in national reports on formulary implementation [5].

Among the recommended medications, lemborexant was the most commonly prescribed medication in the aware group (80%). This may reflect growing evidence that lemborexant carries a lower risk of adverse effects including falls and dependence, particularly in older adults, compared with benzodiazepines and non-benzodiazepine hypnotics [8, 9]. The formulary appears to have effectively disseminated this information to physicians in the awareness group.

Interestingly, no statistically significant differences were found in the overall distribution of commonly prescribed hypnotics between the aware and non-aware groups. This may be due to the increasing mainstream use of orexin receptor antagonists, limitations in sample size, or variability in clinical expertise among respondents.

One of the key implications of this study is that hospital-originated formularies, when effectively disseminated, may provide practical prescribing guidance not only within institutions but also across community healthcare settings. Expanding such formularies into regionally shared tools—so-called “community formularies”—involving collaboration among physicians, pharmacists, and nurses could facilitate more standardized pharmacotherapy. This could lead to improved patient safety and quality of care across the healthcare system. We also recognize that the formulary will require periodic revision. Although the present study evaluated its impact approximately 18 months after introduction, ongoing monitoring will be necessary to assess its continued appropriateness and to guide future revisions.

### Limitations

This study has several limitations. First, voluntary survey responses may have introduced a selection bias, potentially skewing responses toward more engaged or guideline-aware physicians. Second, the overall response rate could not be accurately calculated because the number of physicians who actually received or reviewed the questionnaire was unknown. This uncertainty, along with the fact that respondents were limited to subscribers of the Community Medical Cooperation News, limits the generalizability of the findings. In addition, detailed information on the types of the approximately 1,500 medical institutions to which the questionnaire was distributed was not available, further limiting interpretation of the sample characteristics. Third, although the questionnaire was distributed to institutions, responses were analyzed at the level of individual physicians, and it was unclear whether they reflected institutional or personal views. Fourth, because the formulary was disseminated by a single institution, its broader regional and national applicability remains uncertain. Finally, suggestions for improvements identified in the free-text responses, such as drug-cost concerns and patient communication, require further study and consideration for future formulary development.

## Conclusions

This study demonstrated that, although awareness of the sleep-medication formulary among community-based physicians was low, those who were aware of it were more likely to prescribe the recommended medications and reported significantly less difficulty in selecting an initial hypnotic. These findings suggest that formulary awareness may play a meaningful role in promoting safer and more confident prescribing practices in community healthcare.

Additionally, free-text responses revealed barriers to implementation such as challenges in explaining medication changes to patients and resistance from long-term users of conventional hypnotics. These insights highlight the importance of developing patient-education materials and strengthening interdisciplinary collaboration to support the appropriate use of sleep medications.

To our knowledge, this is the first study to assess both the awareness and practical impact of a sleep-medication formulary on prescribing behavior in a community healthcare context. These findings provide valuable evidence to inform the future design, dissemination, and adoption of community-based formularies aimed at enhancing the safety and consistency of pharmacological treatments across healthcare settings.

## Data Availability

The datasets generated and/or analyzed during the current study are not publicly available due to the inclusion of personal health information but are available from the corresponding author on reasonable request.

## Abbreviations

MHLW: Ministry of Health, Labour and Welfare
OR: Odds ratio
SD: Standard deviation
